# QSAR modeling and molecular docking studies of 2-oxo-1, 2-dihydroquinoline-4- carboxylic acid derivatives as p-glycoprotein inhibitors for combating cancer multidrug resistance^☆^

**DOI:** 10.1016/j.heliyon.2023.e13020

**Published:** 2023-01-20

**Authors:** M. Lahyaoui, A. Diane, H. El-Idrissi, T. Saffaj, Y. Kandri Rodi, B. Ihssane

**Affiliations:** Laboratory of Applied Organic Chemistry, Faculty of Science and Technology, Sidi Mohamed Ben Abdellah University, USMBA, Po. Box 2626 Fez, Morocco

**Keywords:** QSAR, Quinoline, PCA, Machine learning, Deep learning, Molecular docking

## Abstract

Multidrug resistance (MDR) proteins related to the ATP-binding cassette family are found in a very wide range of human tumors and result in therapeutic failure. The overexpression of efflux pumps such as ABCB1 is one of the mechanisms of MDR. This paper aims to develop a reliable quantitative structure-activity relationship (QSAR) model that best describes the correlation between the activity and the molecular structures in order to predict the inhibitory biological activity towards ABCB1. In this regard, a series of quinoline derivatives of 18 compounds were analyzed using different linear and non-linear machine learning (ML) regression methods including k-nearest neighbors (KNN), decision tree (DT), back propagation neural networks (BPNN) and gradient boosting-based (GB) methods. Their aim is to explain the origin of the activity of these investigated compounds and therefore, design new quinoline derivatives with higher effect on ABCB1. A total of 16 ML predictive models were developed on different number of 2D and 3D descriptors and were evaluated using the coefficient of determination (R^2^) and the root mean squared error (RMSE) statistical metrics. Among all developed models, A GB-based model in particular catboost achieved the highest predictive quality, with one descriptor, expressed by R^2^ and RMSE of 95% and 0.283 respectively. Molecular docking studies against the target crystal structure of the outward-facing p-glycoprotein (6C0V) revealed significant binding affinities via both hydrophobic and H-bond interactions with the relevant compounds. The **17** has shown the highest binding energy of −9.22 kcal/mol. Therefore, it can suggest that **17** may prove to be a valuable potential lead structure for the design and synthesis of more potent *P*-glycoprotein inhibitors for combination used with anti-cancer drugs for cancer multidrug resistance management.

## 1 Introduction

All over the world, cancer ranks first among the causes of death [1]. It occurs because of disruption of the physiological functions of cells. A major challenge in developing effective therapy is the resistance of cells to multiple chemotherapy drugs [2]. The ABC (adenosine triphosphate) transporter super family, which transports cytotoxic agents and targeted anticancer drugs using ATP energy, is one of the major mechanisms of drug resistance [3,4,5].

Transporter B1 of the ATP-binding cassette family (ABCB1) is the commonest ABC protein able to mediate multidrug resistance. It is within a system of complex tissue and cellular features that help to bring about drug resistance in cancer cells [6,7,8,9]. In many cases, ABCB1 overexpression is the very first mechanism of resistance, which comes before the development of other mechanisms such as increased drug metabolism, drug target transformation, activation of DNA repair mechanisms, apoptosis checkpoint, and induction of EMT through cell proliferation and the ability to adapt to drug regimens [10]. Pgp (*P*-glycoprotein) has an impact on multidrug-resistant (MDR) cancer and the relationship between Pgp overexpression and MDR cancer has been proven in the literature [11,12,13,14,15,16]. The ability of Pgp to channel such diverse chemical classes is due, in part, to the numerous transport pathways through the protein that have been visualized using molecular dynamics simulations [17]. Work shows that overexpression of Pgp in cancers can be either intrinsic or acquired following drug treatment, depending on the tissue of origin [18].

The biological properties of some quinoline derivatives were found to be interesting and their pharmacological profile advantageous. It was remarked that those compounds with antitumor activity that contain a quinoline moiety perform as cytostatic agents or inhibitors of the topoisomerase-II enzyme, interfering with DNA replication [19,20,21,22,23].

In recent years, machine learning (ML) has drawn the attention of many researchers and has widely been used to develop QSAR models which allow a reliable prediction making of a targeted activity. Several powerful ML algorithms have recently been developed and proven to outperform the most commonly used regression algorithm in QSAR. Many ML models have been developed as each of them inherits a distinct regression algorithm and will therefore provide probable models with different performance. The idea here consists of making a comparative study of these models and choosing the one that performs best and that will guarantee quality predictions. El Hassan El Assiri has used multiple linear regression (MLR) and artificial neural networks to predict corrosion inhibitory activity of pyridazine-derivatives [24,25]. H. El Ghalia has also used MLR to predict the anticancer activity of 5.6.7-trimethoxy-*N*-aryl-2-styrylquinolin-4-amines [26]. Other methods were also used in QSAR modeling such as partial least squares, principal components regression methods and so on.

The molecular docking approach represents a new computational strategy to assess the binding affinity of docked molecules to receptors based on the scoring functions of mathematical algorithms, while the QSAR model produces new compounds with more precise pharmacological efficacy that can serve as effective future drug candidates. Both approaches are considered very effective in silico drug design and can be used separately or simultaneously [27].

The synthesis of novel compounds as potent modulators of ABCB1-induced drug resistance in mouse T-cell lymphoma has been previously reported by Baba, Y. F [28]. in our laboratory. These compounds were assessed for their cytotoxic effect and ABCB1 modulating properties against parental and ABCB1 overexpressing mouse T lymphoma cells. The findings of the rhodamine 123-accumulation assay in multidrug-resistant (MDR) mouse T lymphoma cells overexpressing the ATP-binding cassette B transporter protein will be used to construct QSAR models.

The main objective of this work is to develop mathematical QSAR models describing and predicting the inhibitory activity of quinoline derivatives against ABCB1 from different 2D and 3D molecular descriptors using different new ML regression methods. This is done using the molecular docking study to observe the binding mode of quinoline derivatives with anticancer effect in vitro to the active site of 6C0V, with the aim of synthesizing molecules with the desired biological response before carrying out the experimental synthesis protocol.

## 2 Materials and methods

### 2.1 Data set

**Fig. 1:**
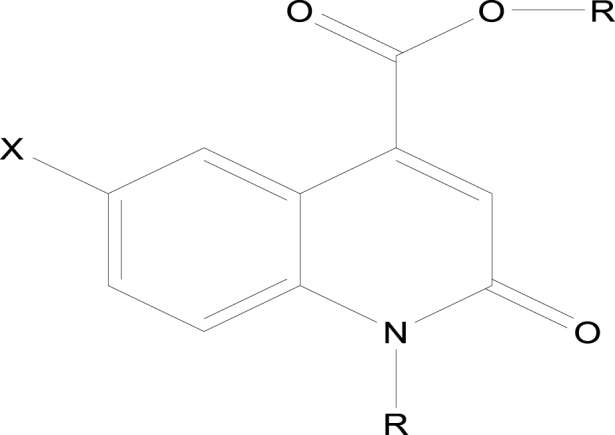
Structure of 2-oxo-1, 2-dihydroquinoline-4-carboxylic acid derivatives.

In this study, 18 derivatives of 2-oxo-1, 2-dihydroquinoline-4-carboxylic acid were prepared by our Laboratory [28], each was synthetized by varying the halogen and the radical (Table 1) of the acid (Fig. 1), for their inhibitory activity against the ABCB1.

**Table 1:** Substituents of 2-oxo-1, 2-dihydroquinoline- 4-carboxylic acid derivatives and their fluorescence activity ratio (FAR).

Compounds	Structure	FAR
1		1.29
2		1.43
3		1.53
4		1.23
5		0.98
6		0.77
7		1.61
8		0.8
9		1.82
10		3.64
11		0.84
12		1.24
13		11.34
14		28.7
15		0.85
16		0.87
17		8.22
18		224.7

**Fig. 2:**
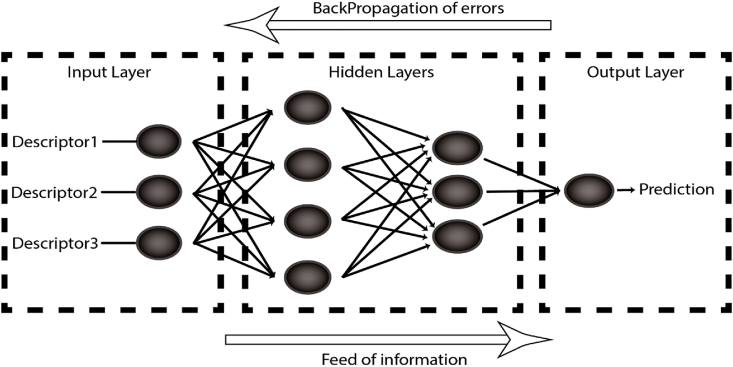
Back propagation algorithm.

### 2.2 Calculation of descriptors

Molecular descriptors are a fundamental element of the QSAR theory, and are in a way the numerical description of a molecular structure. They are used to establish a relationship between the structure of a molecule and its biological activity. In this work, ChemDraw (v.18.2) and Molecular Operating Environment (MOE v.2009.10) were used to draw the molecules and generate 199 2D and 3D descriptors, some of which are shown in Table 2.

**Table 2:** Values of Molecular descriptors.

mol	FAR	b_rotR	dipoleX	lip_acc	logP (o/w)	PEOE_VSA-5	PM3_HF	pmiZ	SlogP	AM1_HOMO	AM1_LUMO	apol	E	mr	vdw_area
1	1.29	0.07	−0.49	4.00	0.96	27.13	−89.97	74.19	1.11	−9.16	−1.18	25.77	13.32	4.98	175.36
2	1.43	0.06	−0.65	4.00	1.59	27.13	−96.03	173.70	1.76	−9.22	−1.29	27.29	13.36	5.46	192.94
3	1.53	0.06	−0.20	4.00	1.79	27.13	−81.68	375.34	1.87	−9.24	−1.35	28.16	12.57	5.71	204.71
4	1.23	0.12	−1.17	4.00	1.42	27.13	−80.80	73.62	1.22	−9.02	−0.97	31.96	35.74	5.96	224.84
5	0.98	0.11	−0.52	4.00	2.05	27.13	−86.95	422.78	1.87	−9.06	−1.15	33.47	34.41	6.45	242.42
6	0.77	0.21	−0.83	4.00	2.10	27.13	−91.81	400.35	2.00	−8.97	−0.92	38.15	32.81	6.91	259.31
7	1.61	0.20	0.00	4.00	2.73	27.13	−97.36	498.44	2.65	−8.97	−1.14	39.66	30.41	7.40	276.89
8	0.80	0.20	−0.14	4.00	2.93	27.13	−81.63	634.13	2.76	−9.03	−1.15	40.53	31.04	7.65	288.66
9	1.82	0.27	−0.09	4.00	3.37	27.13	−45.24	1165.13	2.99	−8.99	−1.19	43.18	35.92	8.23	308.50
10	3.64	0.27	−0.29	4.00	3.58	27.13	−31.30	682.66	3.09	−9.05	−1.19	44.05	36.72	8.48	320.28
11	0.84	0.29	−0.52	4.00	2.03	29.64	29.62	244.48	1.23	−9.04	−1.08	39.00	32.45	7.52	288.08
12	1.24	0.27	−0.22	4.00	2.86	29.64	37.90	887.20	1.99	−9.12	−1.23	41.38	31.63	8.26	317.43
13	11.34	0.19	−0.53	4.00	4.99	27.13	−24.24	1658.82	4.89	−8.97	−1.04	58.42	71.03	10.97	365.84
14	28.70	0.19	−0.86	4.00	5.83	27.13	−15.42	3349.24	5.66	−9.09	−1.13	60.80	70.02	11.72	395.20
15	0.85	0.19	−0.10	6.00	2.45	38.50	−9.46	1434.52	3.68	−8.87	−0.77	55.76	73.39	10.65	362.13
16	0.87	0.19	0.77	6.00	3.08	38.50	−12.54	283.35	4.34	−9.02	−1.23	57.27	73.28	11.15	379.71
17	8.22	0.19	0.62	6.00	3.29	38.50	−0.46	622.57	4.45	−8.99	−1.08	58.14	73.45	11.40	391.48
18	224.70	0.22	0.94	6.00	4.31	24.93	10.66	1109.62	5.26	−9.21	−1.15	58.14	75.45	11.51	387.86

Note: The full name of descriptors can found in annex immediately after the conclusion.

### 2.3 The inhibitory activity response

The rhodamine 123 accumulation assay is a fluorescence detection system that uses verapamil as a reference inhibitor of the ABCB1 efflux pump [29]. The fluorescence intensity of the selected cell population was measured with a Partec CyFlow cytometer (Partec, Munster, Germany). The average fluorescence intensity was calculated for MDR and T-lymphoma cell lines from treated parental mice compared to untreated cells 30,31. The fluorescence activity ratio (FAR) was calculated based on the following equation relating the measured fluorescence values:

FAR=MDRtreatedMDRcontrolPARENTALtreatedPARENTALcontrol

### 2.4 Data split

The data consisted of 18 molecules descriptors was randomly split into training and testing sets. The former consisted of 15 molecules that span the entire chemical space for all the data. While the consisted of three molecules within the Applicability Domain (i.e., the range of the training set).

#### 2.4.1 Data exploration by means of PCA

Principal component analysis (PCA) permits us to verify redundancy and collinearity between the studied descriptors and to carry out a comparative statistical study between the proposed mathematical models such as partial least squares regression (PLS) and stepwise multiple linear regression (SMLR) with the aim of correlating activity with molecular structure [32].

#### 2.4.2 Partial least squares regression

PLS leads to a robust statistical solution when the independent variables are strongly related to each other, or if the independent variables outnumber the observations. PLS is an alternating regression method, which generates its solutions based on the linear transformation of a large number of original descriptors to a small number of new orthogonal terms, called latent variables [33,34]. Therefore, this methodology is considered a standard statistical.

#### 2.4.3 Stepwise multiple linear regression

This approach uses the MLR variant commonly, which generates a multi-term linear equation, although not all of the independent variables are used. This method is well suited to be used when the number of descriptors is large and the main descriptors are unknown [35]. MLR is based on the assumption that the dependent variable is linearly related to some independent variables according to the following relationship.

$$Y={a_{0}}_{+}\sum_{i=1}^{n}a_{iX_{i}}$$

Whereas Y represents the dependent variable (biological activity to be predicted), X_i_ representing the independent variables (molecular descriptors), n indicating the number of molecular descriptors, a_0_ showing the constant in the previous equation, a_i_ being the coefficient of descriptors.

### 2.5 Data modeling

In general, QSAR data consists of a large number of features that reflect physico-chemical properties of molecules and which not all correlate to the target. Moreover, it is often characterized by high redundancy, which frequently leads to instability and increases the variance of ML models. The primary goal of data modeling is to develop prediction models that can accurately describe the FAR target variation based on descriptors that have a high, moderate, or low correlation to it. This helps identify the key property of the molecule that affect its activity, suggest a structure or molecule with a specific activity as well as understand the interaction between functional groups in a molecule. Data modeling was performed, on centered and scaled data, with python core (v3.10) where several machine learning (ML) algorithms were used to develop predictive regression models including, decision tree (DT), k nearest neighbor (KNN), gradient boosting-based (GB) models, back propagation artificial neural network (BPNN) [36,37,38].

#### 2.5.1 Back propagation neural network

Back propagation neural network is a commonly known method for developing predictive models from large datasets. It is a neural network (NN) that is primarily trained through using back-propagation (BP) algorithm. It consists of three sorts of layers (input, hidden, and output layers), each of which is composed of one or more neurons characterized by an activation function and bias (Fig. 2). Neurons in one layer are interconnected to those in the next layer by connections known as synaptic weights, resulting in a network that attempts to mimic the human brain. The BP learning algorithm typically comprises of two successive phases which work to minimize the difference between measured values and the Network output by tuning weights and biases in iterative manner: forward-propagation of information and backpropagation of error. Proper tuning of the weights and biases allows reduce error and make the model reliable. The number of neurons in each layer, appropriate activation functions, the number of layers, the maximum number of iterations. Are all hyperparameters to be tuned in BPNN. In this study, the tuning was performed using a meta-heuristic optimization algorithm called Simulated annealing provided by hyper opt module [39].

#### 2.5.2 K nearest neighbor and decision tree regressors

KNN and DT are two regression methods used in this work because of their simplicity and performance in providing efficient predictive models. They are non-parametric regression methods able to quickly identify the relationship between descriptors and the target. The implementation of DT can also be done without scaling the descriptors and is not largely influenced by the presence of outliers in the data [40].

#### 2.5.3 Gradient boosting algorithms

Gradient boosting is based on the assumption that combining the best next model with the prior models lowers overall prediction errors. Gb algorithms are a family of open-source ensemble methods. They have been widely used for several purposes including, high-precision and adoptable recommender systems building, weather prediction, features selection in regression problems and so on. They allow development of performant and stable predictive models by training a sequence of weak models based on decision trees, each of which compensates for the errors of its predecessors, in contrast to many ML models that concentrate on high quality prediction achieved by a single model. In this work, three GB regression algorithms were used: eXtreme gradient boosting (XGBoost), light gradient boosting machine (LGBM) and categorical boosting (CatBoost) [41].

### 2.6 Predictive models validation and evaluation

After predictive model development, a validation process is required to ensure that the models are performing the way it was intended and that it accurately predict the target. In this work, the validation process was performed in two ways: internal and external. The former was performed using Leave-One-Out Cross Validation (LOOCV) to prevent the model under and over-fitting and perform the hyperparameters tuning. While the later was performed using unseen data and it aimed to test the model capability in making the right predictions in the future. Models evaluation was used to estimate the developed models performance in training, internal and external validation. It was performed using two common statistical metrics: the coefficient of determination (R^2^) and the root mean squared error (RMSE). The best model is characterized with high R^2^, low RMSE and low variance between the train, internal and external validation. Computational formulas are provided in (1(1) and (2)(2):

(1) $$RMSE=\sqrt{\frac{\sum_{i=1}^{N}{\left(ŷi-y¯\right)}^{2}}{N}}$$

(2) $$R^{2}=1-\frac{\sum_{i=1}^{N}{\left(yi-ŷi\right)}^{2}}{\sum_{i=1}^{N}{\left(yi-y¯\right)}^{2}}$$

where yi, ȳ, ŷ, and N are respectively a measured value, the average value of all measured values, the predicted value, the total number of samples.

### 2.7 Molecular docking methodology

The docking process was further investigated between the studied molecules with the best FAR values and the crystal structure of human *P*-glycoprotein in the outward-facing ATP-bound conformation. All scores achieved during the process of molecular docking have been computed and presented using the Molecular Operating Environment (MOE) software. The structures of these compounds were constructed using ChemDraw 18.2 software.

The protein data bank (https://www.rcsb.org/pdb) was used to recover and generate the target crystal structure of outward-facing p-glycoprotein (PDB code = 6C0V). This multi-drug transporter permeability (P)-glycoprotein is a transporter of adenosine triphosphate (ATP) binding cassettes that accounts for clinical resistance to chemotherapy. As such, *P*-glycoprotein extrudes toxic molecules and drugs from cells through ATP-powered conformational changes. To accomplish the optimization, all water-bound cofactors and ligands were detached from the protein structure and the hydrogen atoms were finally attached. The active sites have been sequestered and taken as dummy atoms. The MMFF94*x* force field was adopted to assign all parameters and charges. Following the generation of the alpha site spheres using the MOE site search module, the structural model of the molecules was docked to the surface of the cancer protein interior through the MOE DOCK module. The London dG notation function was used to execute the dock notation in the MOE software and two unrelated refinement methods were then used for the upgrade. Auto-rotating links were then authorized for the top ten link poses that were targeted for analysis to obtain the highest possible score. The database browser was then utilized to match the docking poses to the ligand in the co-crystallized structure along with acquiring the RMSD of the docking pose. Then, the binding free energy and hydrogen bonds between the synthesized molecules and the amino acid residues of the receptors were computed to rank the binding affinity of the molecules to the protein molecules under study. The interaction types together with the RMSD of the (native) ligand in the receptor structure were assumed as the default-docking model.

## 3 Results and discussion

### 3.1 Descriptors exploration

**Fig. 3:**
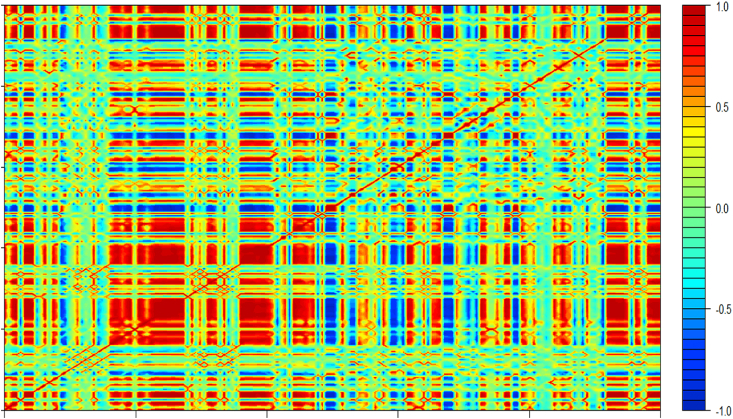
Heatmap of correlation.

A heatmap of correlation shown in (Fig. 3) was used as a tool to provide an overview of the relationship between descriptors. It shows the presence of positive and negative high correlations (red and blue zones which correlation coefficient in absolute error is close to 1); this correlation means that there is a curse of redundancy of information in our data which often leads to the instability of ML models. To overcome this curse, two approaches were used: features extraction by means of partial least squares regression, and feature selection using embedded methods based on random selection. However, the figure also illustrates descriptors which have no significant correlation with any descriptor presented by the sky blue, green and yellow zones (correlation coefficient ranging between −0.5 and 0.5).

### 3.2 Principal component analysis

**Fig. 4:**
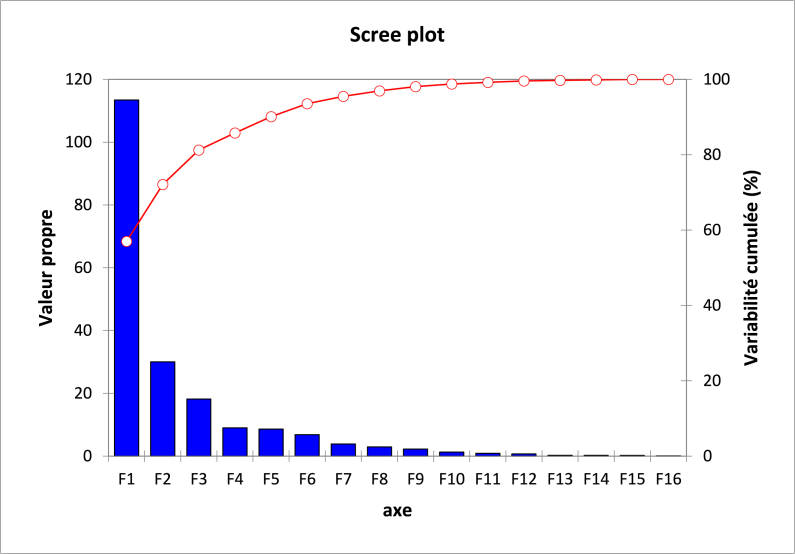
The principal components and their variances.

The PCA was conducted on centered and scaled data in order to identify and select descriptors that correlate to FAR. For this, it was made on 199 descriptors and the sixteen principal components obtained are displayed in Fig. 4.

**Fig. 5:**
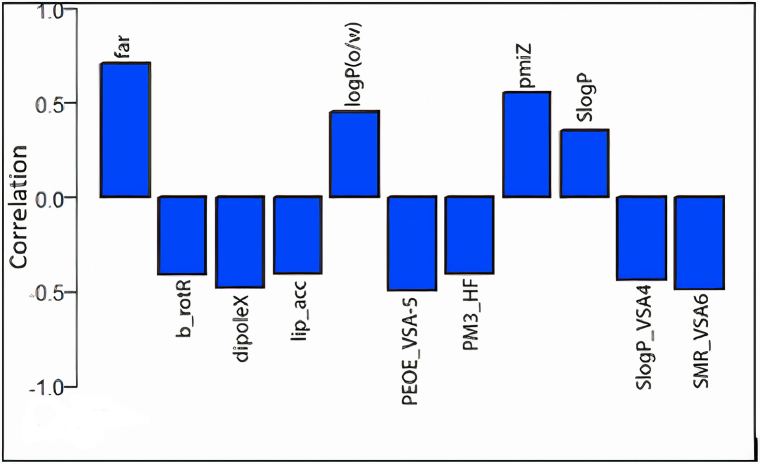
Bar plot of the correlation coefficients between FAR and the most ten correlated descriptors and the principal component that best explain the FAR.

Ten descriptors which have high correlation (Fig. 5) with the component explaining much our target variation were selected to be used for development of SMLR and PLS models.

### 3.3 Partial least squares regression analysis

Partial least squares regression widely used in the case of a high redundancy in the data. It has the ability to distinguish highly informative features from redundant and uninformative ones and therefore helps construct reduced models that retains key features which have a relation with the response.

The resulting PLS model expression, together with the statistical parameter values, is represented by the following equation:

FAR = −3.74 + 2.03 × b_rotR - 1.01 × dipoleX - 0.14 × lip_acc +1.64 × logP (o/w) - 0.04 × PEOE_VSA-5 + 0.01 × PM3_HF + 0.01 × pmiZ + 1.33 × SlogP - 0.01 × SlogP_VSA4 - 0.02 × SMR_VSA6.

**Fig. 6:**
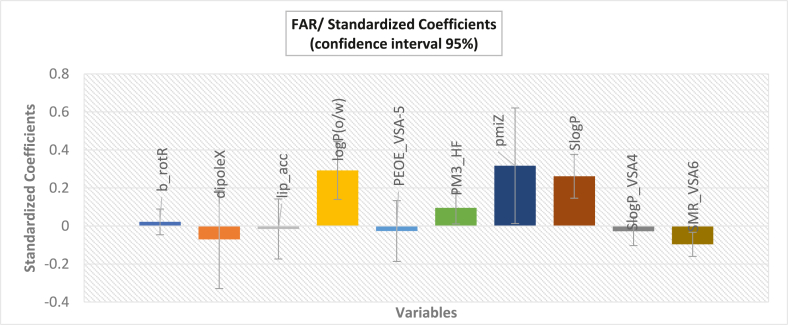
Standardized coefficients versus variables in the proposed PLS model.

Based on the descriptors given in the PLS model equation, the importance of each individual descriptor in relation to the standardized regression coefficients is displayed in Fig. 6.

We observe in Fig. 6, that FAR importance based on molecular structure varies from descriptor to another. Nevertheless, since the descriptors in the PLS model have different units, these standardized coefficients are estimates without real scale, suggesting that these standardized coefficients are not useful for determining the true relative importance and significance of each descriptor in the regression analysis. Furthermore, their value is restricted to determining the positive or negative contribution of molecular indices to the property under study.

**Fig. 7:**
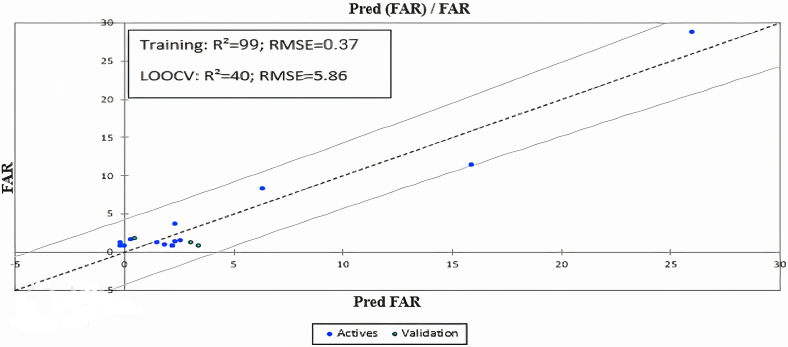
Relationship between the observed Far values and those predicted by the PLS model.

Returning to the statistical parameters and the result obtained in Fig. 7, we see that the model achieved a high performance in the training phase: a high R^2^ value of 99% and a low RMSE value of 0.37. However, the model attained a performance much lower than that obtained in the training (R^2^ = 40%, RMSE = 5.86) which means that it has overfitted the data. The weak statistical results of the PLS model led us to evaluate other statistical models, such as stepwise multiple linear regression and machine learning models.

### 3.4 Stepwise multiple linear regression (SMLR) analysis

Stepwise multiple linear regression is widely regarded as being one of the most fundamental modeling methods recognized in the QSAR field. The ten descriptors resulting from the PCA are an input file for a stepwise selection based on MLR analysis. SMLR consists of treating the links between the dependent quantitative variable to be explained (FAR) and the independent explanatory quantitative variables (descriptors), the expression of the established SMLR model, accompanied with the values of the statistical parameters of five selected descriptors is represented by the following equation:

FAR = 4.51–7.31 × b_rotR - 2.92 × dipoleX + 3.57 × lip_acc +10.44 × logP (o/w) + 4.99 × SMR_VSA6.

**Fig. 8:**
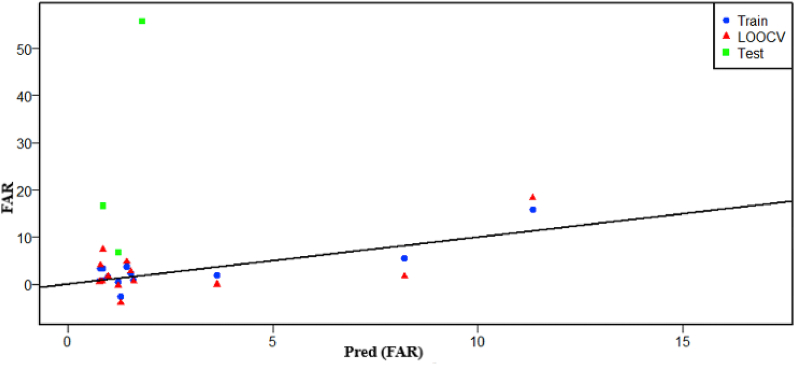
Correlation of FAR and predicted FAR.

The developed model achieved high quality performance during the training phase, expressed by high R^2^ and low RMSE of 90% and 2.377 respectively. However, during the CV the model performance has decreased significantly (R^2^ = 48%, RMSE = 5.325) while during the test the model has not explained any amount of the response variability (R^2^ = 0%, RMSE = 32.546) (Table 3 and Fig. 8). This means it is statistically not acceptable.

**Table 3:** Summary of result model analysis.

	R^2^	RMSE
Train	90%	2.377
CV	48%	5.325
Test	0%	32.546

### 3.5 Machine learning models with feature selection

A summary of developed ML models on different numbers of descriptors (NVAR) is presented in Table 4. It shows that these models have achieved higher performance than SMLR and PLS. in general, most of the models have a good performance in all development and validation phases: training, CV and testing. An objective comparison between them reveals that the KNN (M2), the LGBM (M4), CATBOOST (M8) and BPNN (M15) models outperformed all the other developed models showing simultaneously high predictive performance and low variance through the training and testing phases.

**Table 4:** Summary of developed ML regression models.

Regressor		Model	Train		CV		Test	NVAR
			R^2^	RMSE	R^2^	RMSE	R^2^	
KNN		M1	94	0.316	91	0.394	97	8
		M2	93	0.345	93	0.352	96	2
LGBM		M3	92	0.353	90	0.403	97	8
		M4	94	0.302	93	0.352	97	3
XGBOOST		M5	93	0.332	90	0.397	97	11
		M6	92	0.353	90	0.403	97	159
CATBOOST		M7	95	0.277	91	0.376	96	4
		M8	95	0.283	93	0.346	96	1
DT		M9	92	0.353	90	0.403	97	20
		M10	100	0.076	91	0.387	92	9
BPNN	**1 hidden layer**	M11	94	0.305	91	0.384	95	62
		M12	93	0.342	88	0.437	91	20
	**2 hidden layers**	M13	92	0.354	91	0.392	96	159
		M14	95	0.28	92	0.374	97	20
	**3 hidden layers**	M15	98	0.168	94	0.324	97	24
		M16	100	0.006	93	0.337	70	186

However, a comparison between models with respect to the computational cost and the number of descriptors shows that M15 is not recommended because of its complexity, larger number of descriptors and a higher computational cost. Therefore, the M2, M4 and M8 are the most performant predictive models which can be used for the prediction of biological activity against ABCB1 from a low number of descriptors.

### 3.6 Docking study

**Fig. 9:**
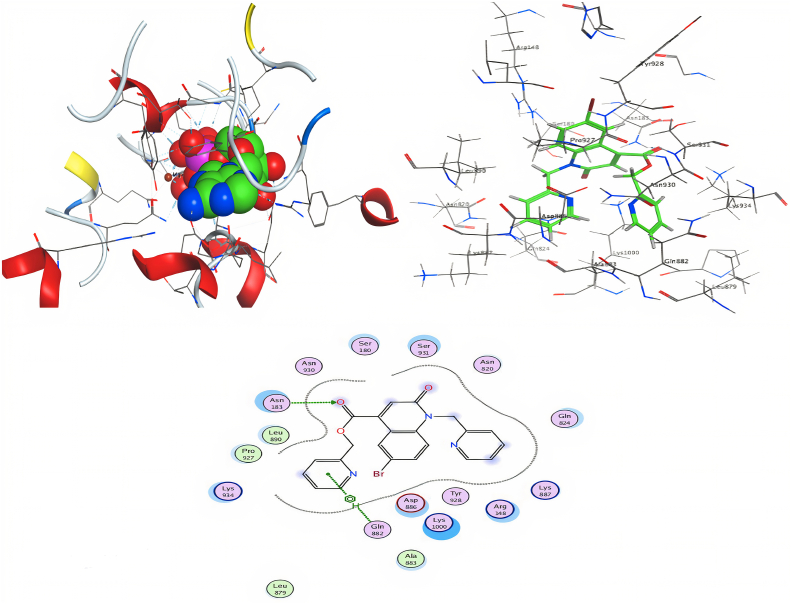
3D docking, site view & 2D of compound 17 and 6C0V protein.

With the intention of targeting more potent molecules, various molecular docking studies were undertaken using MOE software to virtual screen molecular binding modes of four prepared compounds, such as **10**, **12**, **13** and **17**, in the *P*-glycoprotein pocket. Here, the ligands were docked to the encoded protein 6C0V loaded from the PDB. Ten different interaction positions were authorized for each molecule with the protein and the ranking poses were generated by the scoring functions which are given in Table 5. The **17** obtained the highest score, and the result was −9.22 kcal/mol. The list of hydrogen bonds between the compounds and the selected protein coenzymes is given in Table 6. The best-fitting pose that was adopted by the enzyme-calmed compound 6C0V is displayed in Fig. 9.

**Table 5:** Docking score and energy of the compounds and 6C0V protein.

Compound	S	Rmsd_refine	E_conf	E_place	E_score1	E_refine	E_score2
10	−7.905	1.025	1.394	−75.559	−9.623	−18.183	−7.905
	−7.775	0.800	−2.839	−86.373	−9.463	−23.079	−7.775
	−7.645	1.608	−1.793	−49.818	−9.905	−22.728	−7.645
	−7.543	2.688	−0.888	−49.161	−10.039	−22.203	−7.543
	−7.414	2.085	−2.084	−58.509	−9.664	−17.422	−7.414
12	−7.373	1.741	−9.593	−47.854	−9.930	−12.378	−7.373
	−7.297	1.012	−5.187	−79.545	−9.838	−12.151	−7.297
	−7.243	0.885	−12.536	−67.845	−9.334	−6.729	−7.243
	−6.997	1.031	−17.548	−68.131	−9.505	−18.468	−6.997
	−6.652	1.332	−14.182	−84.596	−9.484	−10.754	−6.652
13	−7.947	1.486	36.845	−68.307	−11.132	5.557	−7.947
	−7.789	1.970	43.578	−54.778	−10.129	−8.370	−7.789
	−7.724	1.956	37.138	−72.143	−11.943	−6.981	−7.724
	−7.555	2.616	49.700	−61.442	−10.441	−3.158	−7.555
	−7.129	1.281	37.123	−80.267	−10.301	−1.612	−7.129
17	−9.222	2.289	48.015	−56.777	−11.020	−17.708	−9.222
	−8.637	2.336	37.203	−68.444	−11.376	−8.698	−8.637
	−8.545	2.083	50.419	−62.657	−11.611	−7.534	−8.545
	−8.371	2.000	48.119	−58.199	−11.726	−4.530	−8.371
	−8.057	1.413	48.004	−47.495	−10.832	−4.235	−8.057

**Table 6:** Interaction table between the compounds and 6C0V protein.

Compounds	Ligand		Receptor			Interaction	Distance	E (kcal/mol)
10	O	15	NH1	ARG	148	H-acceptor	3.33	−1
			NH2	ARG	148		2.88	−4.8
	O	25	NE2	GLN	824		2.74	−0.7
			NZ	LYS	1000		2.89	−8.4
12	O	15	ND2	ASN	820	H-acceptor	3.08	−2.8
13	C	13	OD1	ASP	886	H-donor	3.41	−0.8
17	O	21	ND2	ASN	183	H-acceptor	3.17	−1.7
	6-ring		NE2	GLN	882	pi-H	4.18	−1.1

MOE is one of the most important molecular docking mechanisms used to recognize a precise docking study between compounds and target proteins. The compound **17** exhibited a high docking score by hydrogen π-stacking with the 6-membered ring of Gln 882. The interaction distance and stabilization energy were respectively equal to 4.18 A° and −1.1 kcal/mol and by hydrogen bonding of the oxygen atom contained in the ester function to the amino acid residue Asn 183. This hydrogen bond is at about 3.17 A° and the energy stabilization amounts to −1.7 K cal/mol. Through these bonds obtained with the key amino acid residues of the binding pockets highlighted based on the above results, the target receptor structure could be stabilized. The docking pose showing the highest affinity is regarded as the best docking conformation. Similarly, in view of these crucial interactions at the molecular level, MOE was able to match the experimentally observed binding modes, thus identifying the particular conformation of the target and ligand. By comparing the results obtained with the others of biological activity carried out by our laboratory [42], we note that they are the same since the compound **17** that has the best biological activity obtained the best-docked conformation.

## 4 Conclusion

QSAR modeling was performed to develop models for the prediction of the inhibitory effect of quinoline-derivatives on one of the most studied human adenosine-triphosphate (ATP)-dependent efflux transporters that encodes a multidrug resistance protein, called ABCB1 gene. Through this study, several machine learning methods were tested to identify relevant descriptors and develop reliable models including partial least squares regression, stepwise multiple linear regression, back propagation neural networks. The results obtained showed that a catboost model statistically outperformed the other used methods, achieving a R^2^ and RMSE of 95% and 0.28 respectively. Based on the computational study, compound **17** was found to possess the maximum binding affinity to the target protein of outward-facing p-glycoprotein with a binding energy equal to −9.22 kcal/mol. According to the above fact of the laboratory results, this compound can be proposed as a lead structure for the design and synthesis of more potent *P*-glycoprotein inhibitors for combination used with anti-cancer drugs for cancer multidrug resistance management.

Indeed, in this work, descriptors were identified by QSAR models to effectively predict the *P*-glycoprotein inhibitory effect as well as to guide the design of new of 2-oxo 1, 2-dihydroquinoline-4- carboxylic acid derivatives for potential applications in cancer multidrug resistance area. This will help to facilitate the drug development process and minimize the cost of synthesis in pharmaceutical chemistry laboratories.

**Table:** Annex: 1

Abbreviation of molecular descriptors	description
b_rotR	Fraction of rotatable bonds
dipoleX	Dipole moment
lip_acc	Lipinski acceptor count
logP (o/w)	Log octanol/water partition coefficient
PEOE_VSA-5	Total positive surface area
PM3_HF	Heat of formation
pmiZ	Principal moment of inertia
SlogP	partition coefficient
AM1_HOMO	Homo energy
AM1_LUMO	Lumo energy
apol	Sum of atomic polarizabilities
E	Potential energy
mr	Molar refractivity
Vdw_area	Van der Waals surface area

**Table:** Annex: 2

Abbreviation	description
S	The finale score of GBVI/WSA binding free energy
Rmsd_refine	The mean square deviation after refinement
E_place	Score of the placement phase
E_conf	Energy conformer
E_refine	Score refinement
E_scor1	Score the first step of notation

## Author contribution statement

Mouad Lahyaoui: Conceived and designed the experiments; Performed the experiments; Analyzed and interpreted the data; Wrote the paper.

Abderrahim Diane: Performed the experiments; Wrote the paper.

Hafsa El-Idrissi, Taoufiq Saffaj, Ihssane Bouchaib: Performed the experiments.

Kandri Rodi Youssef: Performed the experiments; Contributed reagents, materials, analysis tools or data.

## Funding statement

This research did not receive any specific grant from funding agencies in the public, commercial, or not-for-profit sectors.

## Data availability statement

Data included in article/supplementary material/referenced in article.

The authors declare no competing interests.
